# Treatment of giant intracranial aneurysms: long-term outcomes in surgical versus endovascular management

**DOI:** 10.1007/s10143-022-01884-3

**Published:** 2022-10-21

**Authors:** Antonio Santoro, Daniele Armocida, Francesco Paglia, Marta Iacobucci, Luigi Valentino Berra, Luca D’Angelo, Carlo Cirelli, Giulio Guidetti, Francesco Biraschi, Giampaolo Cantore

**Affiliations:** 1 Rome Human Neurosciences Departmen, AUO “Policlinico Umberto I”, Neurosurgery Division, Sapienza University, Via del Policlinico, 155, 00161 Rome, Italy; 2 Department of Radiological, Oncological and Anatomopathological Sciences, Unit of Interventional Neuroradiology, Sapienza” University of Rome, Umberto I University Hospital, Rome, Italy

**Keywords:** Giant intracranial aneurysm, Neurosurgery, Brain aneurysm, Coiling, Endovascular

## Abstract

Aneurysms with a major diameter > 25 mm are defined as giant intracranial aneurysms (GIAs). Different clinical, pathological, and radiological factors were revealed as playing a role in choosing the best strategy between surgical and endovascular approaches. Despite the improvement of both techniques, the efficacy and safety of these different management are still debated. We evaluated the differences in clinical and radiological outcomes of GIAs treated with surgical and endovascular techniques in a large retrospective mono-centric study. We compared aneurysm location, clinical, morphological features, treatment outcome, and complications on the ground of treatment technique. The final cohort consisted of 162 patients. All the patients were assigned on the ground of the type of eligible treatment: surgical (118 patients) and endovascular procedure (44 patients). The different treatment strategies were made through a multidisciplinary selection whereas clinical parameters, location, and morphologic features of the aneurysm were considered. The surgical group manifested a greater reduction in performance levels and neurological status in the post-operative phases than the endovascular group (*p* < 0.01) with a higher incidence of complications (*p* = 0.012) in contrast to a lower recurrence rate (*p* > 0.01). There is no significant difference in post-operative mortality and survival between surgical and endovascular groups. The surgical group manifested a higher incidence of complications after treatment. The endovascular group has a better post-operative outcome, but a higher risk of recurrence and the necessity of further treatment.

## Introduction

Giant intracranial aneurysms (GIA) are vascular lesions whose diameter is greater than 25 mm ^1^. This uncommon type of lesion represent 5% of all cerebral aneurysms and most frequently involve females in the fifth and seventh decade of life [1, 2]. The natural history of GIAs is characterized by progressive growth, thrombosis, and a high risk of rupture [3, 4]. Clinical presentation of GIA is heterogeneous: 65–85% of patients present with mass effect-related symptoms, 25–35% with subarachnoid hemorrhages (SAHs), ischemic symptoms or stroke, and seizures [5]. If left untreated, mortality rates in patients harboring GIA are around 68–100% at 2 years [6], with an annual risk of rupture around 8–10% [7–11].

These high mortality rates support the indication to treat patients with GIAs to exclude the lesion from the circulation while preserving distal blood flow [12]; although high risks in treatment are recognized, prognostic factors are not precisely known, and there are no specific treatment recommendations for this particular subtype of aneurysm.

Several clinical, pathological, and radiological factors have been assumed to choose the best therapeutic strategy between the surgical and endovascular approaches for GIAs.

From a surgical perspective, different old and new micro neurosurgical techniques are available. We just know a general surgical mortality rate for the treatment of both ruptured and unruptured GIAs that varies between 6 and 22% [10–12].

On the other hand, despite the improvement of endovascular techniques, the efficacy and safety of reconstructive and deconstructive techniques are still not well defined. The appropriateness of treatment is decided on a case-by-case basis considering the high risk of complications and/or recurrence (re-canalization) of the aneurysm [2].

We performed an institutional retrospective review of all patients undergoing treatment for GIA between January 1990 and December 2018 at the Department of Human Neuroscience, Neurosurgical and Neuroradiological Units of Sapienza University in Rome, Italy. The study illustrates our experience with the therapeutic management of GIAs by reporting intra-procedural and delayed complication rates and anatomic and clinical outcomes, concerning treatment modality and vascular characteristics, and highlighting the main elements to consider when considering deciding how to treat this uncommon type of aneurysm.

## Methods

### Patient selection and eligibility

We evaluated all patients with radiological diagnoses of GIA referred to our departments. All patients included in this study met the inclusion and exclusion criteria reported in Table 1; we collected patient demographics, clinical debut, rupture and configuration of GIAs, treatment modality, and outcome. Digital subtraction angiography (DSA), magnetic resonance imaging (MRI), computed tomographic (CT) and computed tomography angiography (CTA), and outpatient clinical data were analyzed independently by a neuroradiologist (M.I) and a neurosurgeon (F.P.).

**Table 1:** Inclusion and exclusion criteria

Inclusion	Exclusion
**•** Patients with radiological diagnosis of an intracranial aneurysm with a diameter of at least 25 mm by means of DSA or computed tomography angiography (CTA), independent of aneurysm shape who underwent surgical or endovascular treatment at our institution;	• History of trauma or iatrogenic injury; • Patients with diseases with poor prognosis (severe heart disease, oncological diseases); • Patients with connective tissue pathology or connetivopathy;
**•** Patients were included in the study if their pre- and post-operative DSA and CT imaging was either performed at our institution or available on the picture archiving and communication system (PACS) for review;	• Patients lost during follow-up (for wrong or incomplete data);
**•** All the patients included in the study were newly diagnosed GIA, at their first surgery;	• Patients untreated;
	• Incomplete or wrong data on clinical, radiological and surgical records
	• Patients with recurrent aneurysms

We retrieved morphological aneurysm characteristics based on vascular origin of the GIA, neck dimension and characteristics, the dome shape and presence of thrombosis.

Patients underwent to a pre-operative protocol of study imaging and selection data reported in repository data of this manuscript.

Clinical follow-up was performed by interventional neuroradiologists (M.I. and C.C.) and neurosurgeons (D.A. and F.P.), depending on the treatment technique. Clinical letters and telephonic questionnaires were used to determine pre-and post-operative functional status, surgical status, and complication rates.

#### Treatment

The goal of the procedures was to exclude the aneurysm from the brain circulation and decompress the nervous structures involved by the dome.

The decision of whether treatment with a micro-neurosurgical rather than endovascular approach was tailored for any patients after a multidisciplinary evaluation with a neuro-interventional radiologist and a neuro-anaesthesiologist considering different variables: aneurysm’s location and morphology of dome and neck, pre-existing pathologies, clinical status (based on Hunt Hess, HH scale and ASA physical status classification system, ASA score), symptoms, and patient’s preference. In general, the patient was not considered eligible to undergo a microsurgical approach in case of massive SAH (with HH grading of 4–5), anesthesiological solid contraindication, and involvement of subtentorial compartments.

#### Surgical procedure

In the surgical cohort (group A), the primary treatment strategy was direct aneurysm neck clipping before thrombectomy, clip reconstruction, or combination with a revascularization surgery. Indirect aneurysm occlusion was performed as an alternative treatment strategy when direct neck clipping was not achievable.

Direct aneurysm clipping was defined as feasible after pre-operative imaging evaluation based on a wide neck or high calcified/absence of a proper neck. We also considered the relationship between the neck and efferent vessels and the location, shape, and fusiform parent artery dilatation. Another essential step for surgical strategy is the tolerance of the balloon occlusion test (BOT). BOT consisted of mechanical occlusion of the cervical portion of the ICA performing clinical observation, and electroencephalographic registration for 30 min. The test was immediately suspended when any neurologic deficit or EEG abnormality was detected.

Three different indirect surgical strategies were performed: (1) trapping of the GIA by distal and proximal occlusion when good collateral blood supply existed in wide-neck GIAs; for those without enough collaterals, a combined revascularization/clipping strategy was performed; (2) proximal occlusion of the cervical ICA if the BOT was tolerated without symptoms; if not, a combined revascularization/clipping strategy was performed; (3) bypass strategy alone. The bypass strategy included the external carotid artery-to-ICA (ECA-ICA) bypass or intracranial internal carotid artery bypass (ICA-ICA). Preferentially GIAs without a recognized neck were submitted to bypass. Only the patients who did not tolerate the pre-operative BOT underwent an ECA-ICA bypass with a spontaneous or an angiographic closure of the ICA within 2 weeks after surgery (staged revascularization) [13, 14].

Exceptions in our series are strongly dependent on intraoperative flowmetry after its introduction in 2001 and the use of a post-operative occlusion test of the ICA.

The staged revascularization strategy included a post-operative occlusion test of the ICA to evaluate the patency of the graft. It confirmed the clinical adaptation of the patient to the new intracranial hemodynamic condition. For all the patients treated microsurgically after 1997, BOT was performed intraoperatively, closing in order the ICA, ECA, and common carotid artery for 15 min during EEG recording to evaluate if the use of the ECA (BOT non-tolerated) or the ICA (BOT tolerated) as donor’s vessel. In the case of ECA-ICA bypass, the ICA was closed intraoperatively after verifying graft patency with intraoperative angiography (single-stage combined revascularization) until 2001 or intraoperative flowmeter (transonic system, Inc., Ithaca, NY) from 2001. In any surgically treated case, intraoperative electrophysiological monitoring (PESS and PEM) was routinely used, intraoperative Doppler ultrasonography, and indo-cyanine green (ICG) fluorescence.

#### Endovascular procedure

Endovascular treatment of giant cerebral aneurysms has changed significantly during the last decades as many new technological advances were introduced:

### Vessel sacrifice with balloon test occlusion

Proximal occlusion of GIAs via endovascular parent vessel sacrifice is feasible when appropriate collateral circulation exists proved by BOT. To avoid recurrences, it is essential to have complete occlusion of the neck of the aneurysm to exclude the possibility of recanalization through collaterals activation, like in posterior communicating artery aneurysms. However, patients who pass BOT still have a 5–25% risk of ischemic stroke after parent vessel sacrifice secondary to hemodynamic and thromboembolic causes [15]. We generally recommend the vessel sacrifice for an older patient who tolerates BOT and compressive symptoms well.

### Coil-assisted embolization

Coil embolization alone for unrupted GIAs is no longer an acceptable strategy for their management. This technique is associated with significantly high re-treatment rates. Re-canalization is highest in the setting of wide residual aneurysm necks, mainly due to coils compaction, growing residual aneurysm neck, and refilling fundus [16, 17]. Moreover, the necessity of dual antiplatelet therapy limits the use of these devices in the acute setting. We reserved treatment with coiling alone to the few patients (6 patients, 13.6%) with severe contraindication to surgery but who displayed a good health status without SAH, with supratentorial GIA with the presence of neck in the absence of apparent thrombosis. Coil embolization was assisted by a vascular reconstruction device, such as Neuroform Atlas Stent (Stryker Neurovascular, Fremont, California, USA) or Low-profile Visualised Intraluminal Support (LVIS; Microvention, Tustin, California, USA); these were designed to augment coil embolizations of wide-neck aneurysms. Both LVIS Jr and Neuroform Atlas stents provide low-profile advantages with delivery through a 0.017-inch microcatheter. Open cells stent could be used in a Y- or X-stent configuration to maximize the coils packing density.

### Flow diversion embolization

In this series, the Pipeline Embolisation Device (PED; Medtronic Neurovascular, Irvine, California, USA), Silk (Balt, Montmercy, France), The Flow Re-Direction Endoluminal Device (FRED, Microvention, Tustin, California, USA), and Derivo flow diverter (Acandis Pforzheim Germany) were used. They have been used alone or in combination with coiling. This is to minimize the risk of post-procedural aneurysmal rupture. All the patients treated with flow diverter (FD) receive 5 days of dual antiplatelet therapy before the intervention. The necessity of dual antiplatelet therapy limits the use of these devices in the acute setting.

We usually provide a staged treatment for ruptured GIAs with SAH, coiling in the acute phase, and flow diverter treatment following recovery from SAH. This plan is safe and effective, as demonstrated in a recent series [18], where no cases of re-bleeding occurred during the interval between coiling and flow diversion. Carotid or vertebral artery occlusion in the acute phase of SAH cannot be recommended because vasospasm may develop, and ischemic events may be aggravated by diminished reserve capacity after occlusion of the artery.

### Statistical methods

The sample was analyzed with SPSS version 18. A comparison between ordinal and nominal variables was made with the ANOVA test, and a comparison between two nominal variables was created with the Chi^2^ test. Pre-operative and post-operative study outcomes were examined with analysis of variance and repeated measures general linear models adjusted for age and sex. Analyses were based on 2-sided tests with values of *p* < 0.05 considered significant with Bonferroni correction when appropriate. Data reported in the study have been completely anonymized. No treatment randomization has been performed. This work is part of a retrospective arm of a study approved by our institution’s ethics committee with number IRB 6168 Prot. 0935/2020. All procedures performed in studies involving human participants were in accordance with the ethical standards of the institutional and/or national research committee and with the 1964 Helsinki declaration. This article does not contain any studies with animals performed by any of the authors. Informed consent was obtained from all individual participants included in the study.

## Results

The clinical, radiographic characteristics, treatment methods, and outcomes of our GIAs series are summarised in Tables 2, 3, 4. The final cohort consisted of 162 patients (Table 2). Of these patients, 99 were females (61.1%) and 63 males (38.9%). The sample age showed a normal distribution after applying the Lilliefors correction with a mean age of 49.53 years old (median 51, SD 14.07); the treatment modality was for 118 patients (72.8%) a surgery, and 44 (27.2%) performed an operative DSA.

**Table 2:** Demographic and clinical data of the examined population

Total patients no. 162	
Sex (female)	99 (61.1%)
Age	49.53 years (median 51, SD 14.07)
Localization	Posterior circulation: 16 (9.9%)
	ICA petrous: 3 (1.9%)
	ICA cavernous: 41 (25.3%)
	ICA clinoid/supraclinoid: 53 (32.7%)
	ICA bifurcation: 11 (6.8%)
	MCA: 23 (14.2%)
	ACoa: 15 (9.3%)
Types	Saccular: 115 (71%)
	Fusiform: 27 (16.7%)
	Dissecting: 20 (12.3%)
Neck characteristics	No proper neck: 34 (21%)
	Neck > 4 mm: 40 (24.7%)
	Neck > 4 mm: 40 (24.7%)
	Calcified Neck: 12 (7.4%)
Intra-aneurysmal thrombosis	No thrombosis: 116 (71.6%)
	Thrombosis < 50%: 42 (25.9%)
	Thrombosis > 50%: 4 (2.5%)
Clinical debut	Mass effect/cranial nerve compression: 75 (46.3%)
	Headache: 13 (8%)
	TIA: 17 (10.5%)
	Seizure: 12 (7.4%)
	SAH symptoms: 33 (20.4%)
	Incidental diagnosis: 11 (6.8%)
Hunt-Hess scale	Grade 1: 123 (75.9%)
	Grade 2–3: 23 (14.2%)
	Grade 4–5: 16 (9.9%)
Modified Rankin Scale at first evaluation	< 2: 111 (68.5%)
	> 2: 51 (31.5%)
Treatment modality	Surgery: 118 (72.8%)
	Endovascular: 44 (27.2%)

**Table 3:** Clinical and radiological analysis between surgical (group A) and endovascular (group B) groups of patients

162 pts	Group A Surgical series, 118 pts	Group B Endovascular series, 44	*p***-**value
Mean age	47.87 min = 18 max = 72 SD = 10.59	53.98 min = 10 max = 86 SD = 20.2	
Female	60/118, 50.8%	39/86, 88.6%	< 0.05
Location	Posterior circulation, 8 pts, 6.8%	Posterior circulation, 8 pts, 18.2%	
	ICA petrous, 3 pts, 2.5%	ICA petrous, 0%	
	ICA cavernous, 25 pts, 21.2%	ICA cavernous, 16 pts, 36.4%	
	ICA clinoid/sovraclinoid, 49, 38.1%	ICA clinoid/sovraclinoid, 19, 43.2%	
	MCA, 22 pts, 18.6%	MCA, 1 pt, 2.3%	
	AcoA, 15 pts, 12.7%	AcoA, 0%	
Neck	No proper neck, 30 pts, 25.4%	No proper neck, 4 pts, 9.1%	0.02
	Neck < = 4 mm, 65 pts, 55.1%	Neck < = 4 mm, 11 pts, 25%	0,01
	Neck > 4 mm, 11 pts, 9.3%	Neck > 4 mm, 29 pts, 65.9%	< 0.01
Neck calcified	12, 10.2%	0%	0.19
Shape	Dissected = 0%	Dissected = 20, 45.5%	< 0.05
	Saccular = 93, 78.8%	Saccular = 22, 50%	0.07
	Saccular = 93, 78.8%	Saccular = 22, 50%	< 0.05
Thrombosis	< 50% = 34, 28.8%	< 50% = 12, 27.3%	0.51
	> 50% = 2, 1.7%	> 50% = 2, 4.5%	0.3
	No thrombosis = 84, 71.2%	No thrombosis = 32, 72.7%	0.51
Debut	Incidental = 10, 8.5%	Incidental = 1, 2.3%	0.15
	SAH symptoms = 28, 23.7%	SAH symptoms = 5, 11.4%	0.06
	Seizure = 12, 10.2%	Seizure = 0%	0.02
	TIA/ischemia = 17, 14.4%	TIA/ischemia = 0%	0.03
	Headache = 0%	Headache = 13, 29.5%	< 0.01
	Mass effect/focal deficit = 50, 42.7%	Mass effect/focal deficit = 25, 56.8%	0.07
MRS pre-op	< 2 = 67, 56.8%	< 2 = 100%	< 0.01
	> 2 = 51, 43.2%		
Hunt-Hess	HH 3–4 = 7, 5.9%	HH 3–4 = 9, 20.5%	< 0.01
	HH 1–2 = 22, 18.6%	HH 1–2 = 1, 2.3%	0.004
	HH 0 = 89, 75.4%	HH 0 = 34, 77.3%	0.49

**Table 4:** The different approaches and outcome analysis

162 pts	Surgical series, 118 pts	Endovascular series, 44	*p*-value
Approach	Trapping = 2, 1.7%	Stent + flow diverter = 1, 2.3%	
	Hunterian ligature = 2, 1.7%	Coiling + stent = 3, 6.8%	
	Clipping = 67, 56.8%	Flow-diverter = 12, 27.3%	
	ICA-ICA bypass = 27, 22.9%	Coiling + Flow-diverter = 16, 36.4%	
	ECA-ICA bypass = 20, 16.9%	Coiling = 6, 13.6%	
		Carotid closure = 13.6%	
Performance status (MRS)	Post-op = < 2 = 56, 47.5%	Post-op = < 2 = 38, 86.4%	** < 0.01**
	> 2 = 61, 51.7%	> 2 = 6, 13.6%	
	3 months = < 2 = 94, 79.7%	3 months = < 2 = 38, 86.4%	0.18
	> 2 = 16, 13.6%	> 2 = 3, 6.8%	
Mortality	Post-op = 1, 0.8%	Post-op = 0%	0.73
	1 month = 1, 0.8%	1 month = 3, 6.8%	**0.02**
	3 months = 7, 5.9%	3 months = 0, 0%	0,116
	Last evaluation = 8, 6.8%	Last evaluation = 3, 6.8%	0,616
Complications	After procedure 47, 39.8%	After procedure 8, 18.2%	**0.01**
	After 1 year 17, 14.4%	After 1 year 0%	**0.02**
Re-habitation	13, 11%	9, 20.5%	**0.01**

### Analysis of groups

All the patients who met the inclusion mentioned above and exclusion criteria were assigned on the ground of treatment: surgical or endovascular procedure (respectively group A and group B). Group A consisted of 118 patients (mean age: 47.87 years, range 18–72); group B consisted of 44 patients (mean age 53.98 years, range 10–86). The average age was found to be comparable between the two test groups. The morphological and clinical characteristics of the two different groups analyzed are presented in Table 3.

The choice to treat a GIA by the endovascular route rather than transcranial surgery was made through a multidisciplinary discussion. In contrast, clinical parameters, location, and morphologic features of the aneurysm were considered. We recognized that the endovascular strategy was the best treatment modality for posterior circulation GIAs, selected in the most of cases as first choice.

The second factor on which the choice was based was the clinical parameters: GIA with acute clinical onset as symptomatology related to endocranial hypertension, ESA (28 pts (23.7%) vs. 5 pts (11.4%), *p* = 0.06), seizure (12 pts (10.2%) vs. 0% *p* = 0.02), and ischemia (17 pts (14.4%) vs. 0%, *p* = 0.03) with pre-operative MRS more impaired (*p* = 0.01) from the functional and performance point of view, were more frequently selected for endovascular treatment (details in Table 3).

In anterior healthy GIAs, aneurysms with unidentifiable necks, with necks less than 4 mm in size, or calcified necks were more frequently selected for surgery (*p* = 0.01).

The presence of endoaneurysmal thrombosis appears to be no significantly distributed among the selected procedures (group A, < 50% = 34–28.8%; > 50% = 2–1.7; no thrombosis = 84–71.2% group B, < 50% = 12–27.3%; > 50% = 2–4.5%; no thrombosis = 32–72.7% *p* = 0.51 *p* = 0.3 *p* = 0.51 respectively). This variable was taking in account for the selection of appropriate endovascular treatment.

### Angiographic and clinical outcomes

**Fig. 1:**
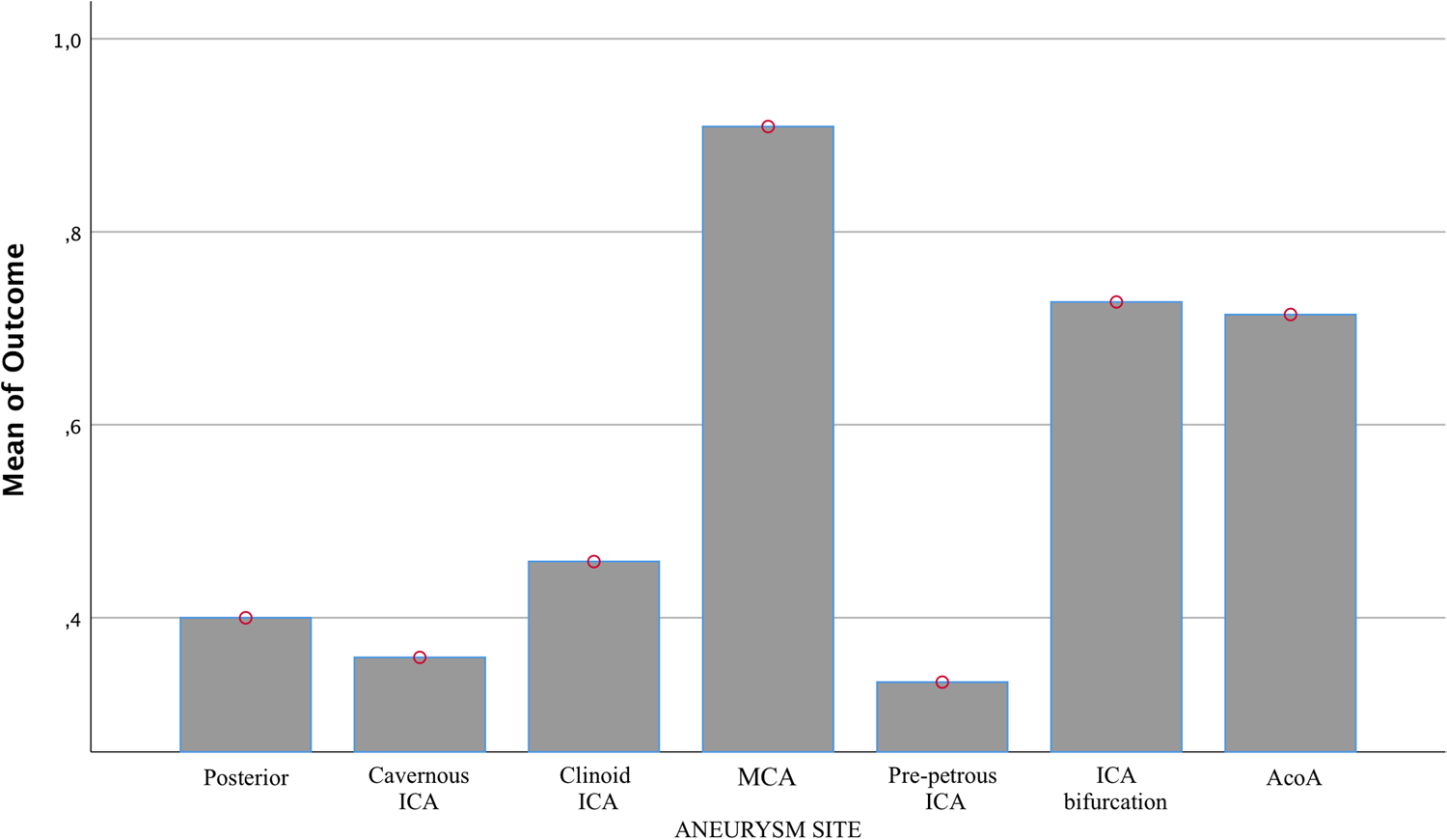
The bar graph of the ANOVA study shows the outcome difference on the ground of GIA’s aneurysm site. The bars show the percentages of cases in which a better clinical outcome was obtained, showing a significant difference in patients in which the GIA was localized at the MCA, the bifurcation of ICA, and at the AcoA complex

In general, there is a significant correlation between survival and the location of GIAs. More favorable localizations are identified for aneurysms originating from MCA, AcoA, and carotid bifurcation (*p* = 0.01, Fig. 1).

**Fig. 2:**
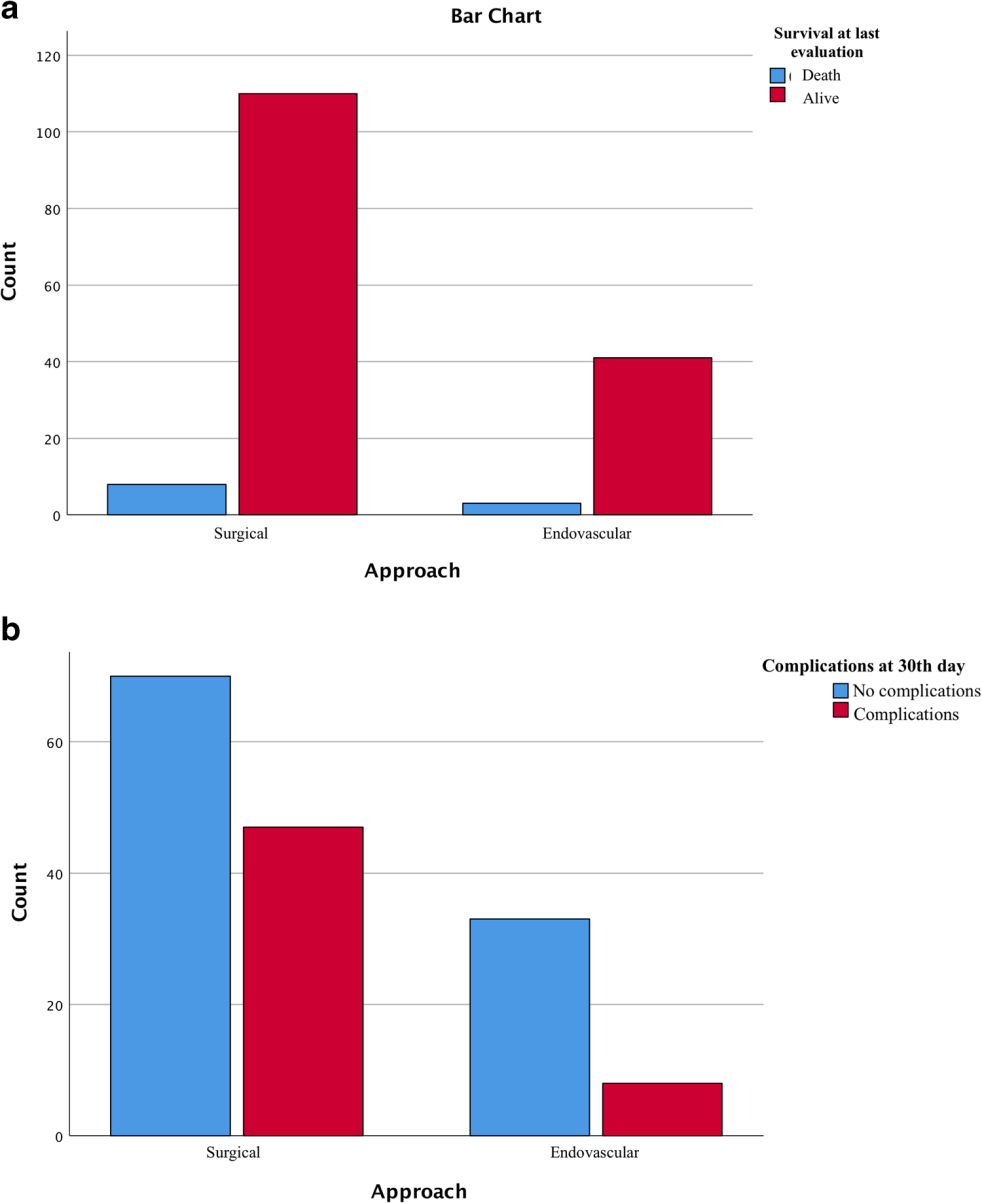
Bar chart with chi-square analysis shows a not significant difference in overall survival between the two groups (part **A**) and a substantial difference in complications in the surgical group after the 30th day (part **B**)

Observing the prospective outcome in patients shows that there is no significant difference in post-operative mortality (in the first 24–48 h) between the two groups (*p* = 0.73); in the individual outcome measures, it seems there is higher mortality found in the first 30 days after the procedure in the endovascular group; at the last evaluation, the difference in overall survival is not significant (*p* = 0.616, Fig. 2A).

Group A manifested a more significant reduction in performance levels and neurological status in the post-operative phases than group B (*p* < 0.001); this finding is confirmed in part by the higher incidence of complications at the last evaluation (respectively *p* = 0.01 and *p* = 0.02 Fig. 2B) in the surgical series than in the endovascular series.

**Fig. 3:**
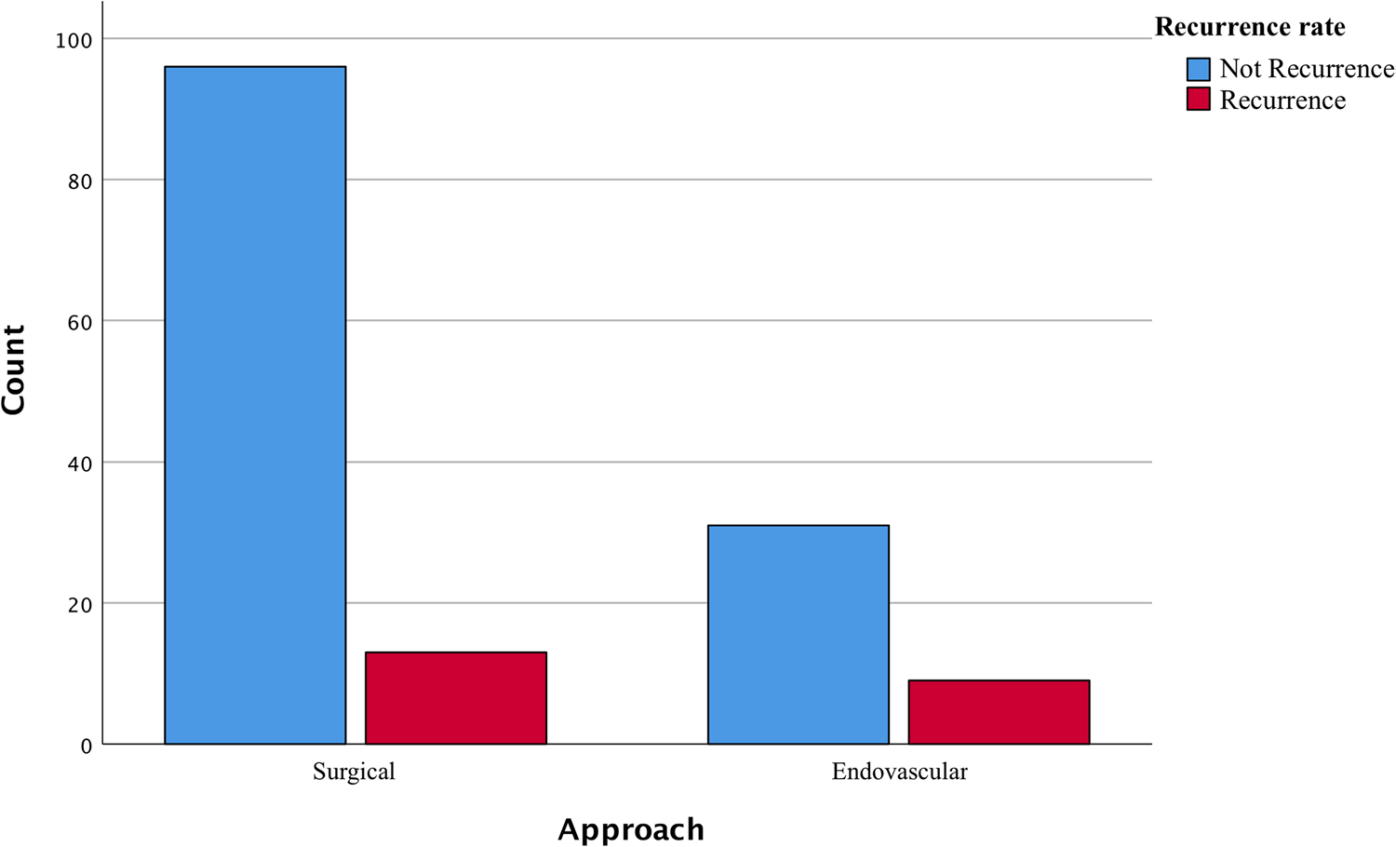
A chi-square analysis demonstrates a valuable difference in the percentage of recurrence of the aneurysm after one year in the endovascular group

From an outcome perspective, it is interesting to note the presence of a lower rate of aneurysm recurrence (intended as re-canalization of dome) in the surgical group compared with the endovascular group (*p* < 0.01 Fig. 3).

Analyzing the different rates of recurrence, complications, and mortality, no significant differences were found in other treatment modalities in group B (*p* = 0.1, ANOVA study).

The same findings were retrieved for group A with the difference that a high rate of late complications is present in GIAs treated with bypass without differences in mortality rate (*p* < 0.01, ANOVA study). Data of outcome are summarized in Table 4.

**Fig. 4:**
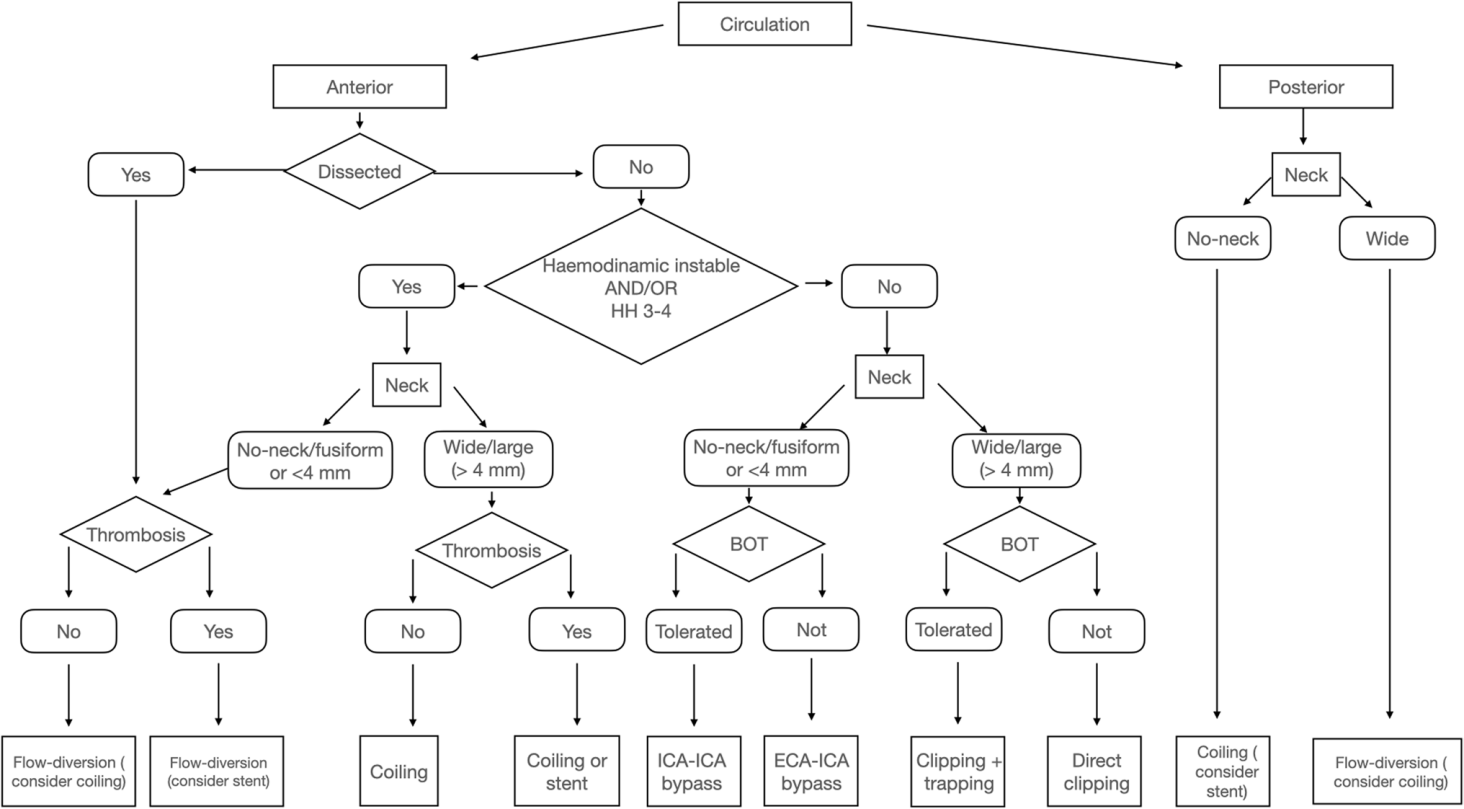
The algorithm summarizes the criteria chosen to select the best treatment for the GIAs proposed in this study

Based on the best results obtained in consideration of the variables that were deemed most important for the choice of treatment that in the results we set forth, we finally designed a treatment algorithm (Fig. 4).

## Discussion

The treatment of GIAs represents a significant challenge among vascular lesions. Although the continuous improvement of neurosurgical and endovascular techniques, no treatment is considered a gold standard [19]. If indeed by now the endovascular procedure is becoming the first choice of treatment for most intracranial aneurysms, the same cannot be said for GIAs [20]. We show an adequate and comparable result between the two strategies, with an acceptable percentage of survival and complication rate (6.8% of mortality and an mRS < 2 in 79.7% of patients after 3 months) reported in the largest published series [21–23], for the surgical group, and comparable results for the endovascular group (6.8% of mortality and an mRS < 2 in 86.4% of patients after 3 months), where different series [24–27] show a rate of mortality and morbidity between 3.8% and 9%.

In literature, few studies of GIAs compare the outcomes of the two different treatment strategies; in some collections [11, 23, 28–30], it is argued that a different rate of complications and obliteration rates among reconstructive techniques for each strategy. On other hand, designing treatment strategies for GIAs is difficult as evidence of large clinical trials is lacking [31–33].

In our series, we did not find significant prognostic differences between approaches. The most evident difference lies in a different total number of complete occlusion between the two strategies in front of the same good outcomes.

Given these promising results in terms of outcome between the two groups, we tried to analyze the variables taken into account to choose the most appropriate treatment.

According to the current epidemiology [31, 32], we found GIAs located more frequently in the anterior circulation. Here we have recognized that, regardless of treatment modality, there is a significant difference in outcome concerning the aneurysm site, where localizations such as MCA, AcoA, and the supraclinoid portion carotid artery pose a lower risk of mortality. We confirmed [33, 34] that when GIAs appear in the posterior circulation, the complications are more severe, and they have a worse outcome [35] since the brainstem has a functional role in the control of essential body functions such as breathing, swallowing, heart rate, blood pressure, and consciousness [8]. The anatomic locations of GIAs adjacent to the brainstem increase the difficulty of surgical treatment, and patients with this localization are more frequently selected for endovascular intervention in our series, where its evaluation was posed as the first factor evaluated.

Patient factors (age, comorbidity, clinical condition) [36], pathological factors (aneurysm location, morphology) [31], and team experience [5] play a crucial role in the decision-making for GIAs.

In addition to size, we recognized that what makes these lesions so complex is the morphology of the aneurysm, the presence of intraluminal thrombus, calcifications of the aneurysmal wall, and the presence or absence of collateral vessels, and location [37].

About morphological type of aneurysm, three major conformations have been described: saccular, fusiform, and serpentine/dissected. Saccular GIAs are thought to develop from smaller saccular aneurismatic lesions due to wall remodeling caused by high hemodynamic stress and blood flow that could increase the risk of intra-operative rupture when assumed a serpentine or dissected conformation [2]. For these reasons, the endovascular approach is generally preferred for dissected GIAs. We have placed the location and morphology of the aneurysmal wall as the foremost factors for treatment choice because they respond best to endovascular treatment regardless of the patient’s clinical characteristics at diagnosis. The clinical and neurological status are among the first variables to consider when making the diagnosis of a non-dissected supratentorial GIA. We suggest to consider surgery as the first choice in case of good health conditions of patients with good HH grades. In surgery, new techniques are available today, although direct occlusion with clipping is the technique of choice [21], reporting percentages of occlusion of 100% [9] and decompression of surrounding parenchyma. There are different methods for clipping, such as tandem or parallel clipping, combined clipping using a fenestrated and straight clip, and dome clipping. In some cases, temporary trapping, thrombectomy, or clip reconstruction may be required, especially with wide-necked aneurysms [25, 26, 38]. Based on our experience, the most critical surgical factor for the technical decision is the characteristics of the neck: in the case of an absent or small channel (defined as < 4 mm [39, 40]) or in surgical cases of fusiform aneurysms, the first alternative technique taken into consideration is the bypass (that can be high-flow extracranial-intracranial bypass, low-flow bypass with superficial temporal artery or reimplantation/reanastomosis [31, 41], based on BOT).

Suppose there is a calcified aneurysm or a thrombus in the neck or perforating vessels arising from the dome, in that case, other alternative techniques can be used, such as wrapping, trapping, and aneurysmectomy. BOT should precede the procedure in which there are parent vessels sacrificed [42–45].

Considering the endovascular perspective, the introduction of FD devices has revolutionized the management of giant, wide-necked, or dissecting/fusiform intracranial aneurysms [25, 46]. FD shows a higher obliteration rate than stent-assisted coiling in unruptured supratentorial GIAs, whereas coiling alone should be preferred for posterior circulation and ruptured lesions with a wide neck.

Among coiling techniques [25, 47, 48], it has been shown that the results obtained with coiling alone or stent-assisted coil are not as effective due to the risk of re-canalization and migration of coils [49, 50]. However, there is a slight difference in the percentage of re-habitation in our series. Furthermore, in various cases, combining FD with coil embolization should accelerate the aneurysmal sac’s thrombosis, which suggests that these two techniques should be considered complementary rather than competitive [24].

In conclusion, according to the literature (advanced by recent meta-analysis and series [22]), we can affirm that although the endovascular approach is nowadays considered a well-accepted technique even for GIAs, microsurgery shows advantages that make it still the first-line treatment in most cases [9, 10, 17, 51]. For their essential mass effect, the goal of therapy is both the occlusion of the aneurysmal sac and the reduction of this effect on the brain [52, 53], which makes endovascular treatment unacceptable in most cases. In contrast, some authors believe that given the risks associated with microsurgery and the less-invasive endovascular approach, the latter should be applied [38, 54–58].

## Limitations and further study

Our study has several limitations; the study was a retrospective study over a long period, which might include some confounding factors, such as patient selection and treatment technique bias. GIAs is a rare disease, and collecting a large case series presupposed a review of cases spanning almost three decades. In the early 1990s, endovascular treatments were less frequent and not equipped with current technology. Another limitation is that being a single-center study, surgical indications, procedures, and peri-operative patient management may vary according to institutional guidelines and experience.

## Conclusion

The mortality rate is comparable between the endovascular and surgical groups regarding the treatment of GIAs. Both endovascular and surgical treatment could be safe and effective and lead to favorable outcomes for GIAs. Endovascularly treated patients have a better post-operative outcome and a lower rate of complications but a higher risk of recurrence and necessity of further treatment. Despite the improvement of the various techniques, the efficacy and safety are still unclear, and the appropriateness of the treatment is decided case by case, preferring high-risk patients, posterior location, or dissecant GIAs for the endovascular procedure and healthy, young, supratentorial, and wide neck GIAs for surgery.

## Abbreviations

GIA: Giant intracranial aneurysms
SAHs: Subarachnoid hemorrhages
EC-IC: External carotid-intracranial carotid bypass
FD: Flow diverters
MRI: Magnetic resonance imaging
ICA: Internal carotid artery
VBS: Vertebrobasilar system
CTA: Computed tomography angiography
MCA: Middle cerebral artery
ACA-ACoA: Anterior cerebral-anterior communicating
BTO: Balloon test occlusion
TIA: Transitory ischemic attach
FD: Flow diverter
